# Comparison of gray matter volume between migraine and “strict-criteria” tension-type headache

**DOI:** 10.1186/s10194-018-0834-6

**Published:** 2018-01-15

**Authors:** Wei-Ta Chen, Kun-Hsien Chou, Pei-Lin Lee, Fu-Jung Hsiao, David M. Niddam, Kuan-Lin Lai, Jong-Ling Fuh, Ching-Po Lin, Shuu-Jiun Wang

**Affiliations:** 1 Department of Neurology, School of Medicine, National Yang-Ming University, Taipei, Taiwan; 2 The Neurological Institute, Taipei Veterans General Hospital, Taipei, Taiwan; 3 Brain Research Center, National Yang-Ming University, Taipei, Taiwan; 4 Institute of Brain Science, School of Medicine, National Yang-Ming University, Taipei, Taiwan; 5 Institute of Neuroscience, National Yang-Ming University, Taipei, Taiwan; 6 Department of Biomedical Imaging and Radiological Sciences, National Yang-Ming University, Taipei, Taiwan

**Keywords:** Tension-type headache, Migraine, Gray matter, Voxel-based morphometry, Magnetic resonance imaging (MRI)

## Abstract

**Background:**

Despite evidently distinct symptoms, tension-type headache (TTH) and migraine are highly comorbid and exhibit many similarities in clinical practice. The purpose of this study was to investigate whether both types of headaches are similar in brain morphology.

**Methods:**

Consecutive patients with TTH and age- and sex-matched patients with migraine and healthy controls were enrolled for brain magnetic resonance imaging examination. Patients with TTH were excluded if they reported any headache features or associated symptoms of migraine. Changes in gray matter (GM) volume associated with headache diagnosis (TTH vs. migraine) and frequency (episodic vs. chronic) were examined using voxel-based morphometry. The correlation with headache profile and the discriminative ability between TTH and migraine were also investigated for these GM changes.

**Results:**

In comparison with controls (*n* = 43), the patients with TTH (25 episodic and 24 chronic) exhibited a GM volume increase in the anterior cingulate cortex, supramarginal gyrus, temporal pole, lateral occipital cortex, and caudate. The patients with migraine (31 episodic and 25 chronic) conversely exhibited a GM volume decrease in the orbitofrontal cortex. These GM changes did not correlate with any headache profile. A voxel-wise 2 × 2 factorial analysis further revealed the substantial effects of headache types and frequency in the comparison of GM volume between TTH and migraine. Specifically, the migraine group (vs. TTH) had a GM decrease in the superior and middle frontal gyri, cerebellum, dorsal striatum, and precuneus. The chronic group (vs. episodic group) otherwise demonstrated a GM decrease in the bilateral insula and anterior cingulate cortex. In receiver operating characteristic analysis, the GM volumes of the left superior frontal gyrus and right cerebellum V combined had good discriminative ability for distinguishing TTH and migraine (area under the curve = 0.806).

**Conclusions:**

TTH and migraine are separate headache disorders with different characteristics in relation to GM changes. The major morphological difference between the two types of headaches is the relative GM decrease of the prefrontal and cerebellar regions in migraine, which may reflect a higher allostatic load associated with this disabling headache.

**Electronic supplementary material:**

The online version of this article (10.1186/s10194-018-0834-6) contains supplementary material, which is available to authorized users.

## Background

Tension-type headache (TTH) and migraine are both common headache disorders. In a Danish population survey, the prevalence of migraine did not change significantly (11–15%) during a 12-year period, whereas the prevalence of TTH (79–87%) increased significantly and was higher than that of migraine [1]. In the Global Burden of Diseases, Injuries, and Risk Factors Study 2016, tension type headache ranks third in terms of global prevalence, and sixth in terms of global incidence, both being higher than that of migraine [2]. Despite the high prevalence and incidence of TTH, scientific interest in TTH has long been sparse probably because the pain intensity, associated symptoms and functional impairment caused by TTH are relatively mild compared with those caused by migraine.

According to the International Classification of Headache Disorders, third edition beta (ICHD-III beta) [3], the characteristics of TTH and migraine are undeniably distinct. Migraine headache is typically unilateral, pulsatile, moderate-to-severe in intensity and aggravated by daily activities. By contrast, TTH is bilateral, non-pulsatile, and mild-to-moderate in intensity and not aggravated by daily activities. In clinical practice, however, TTH and migraine have many similarities, including common triggers, psychiatric comorbidities and responsiveness to similar medications [4]. Moreover, migraine and TTH usually coexist in the same patient [1], which may result in individuals with TTH being clinically diagnosed and treated as more disabling migraine. In the Spectrum Study, 37% of patients initially diagnosed with TTH were later revealed to have migraine or migrainous headache [5]. A recent study using taxometric analysis did not support that TTH and migraine are separate clinical entities [6]. In pathophysiology, both types of headaches have been characterized by central sensitization, as revealed in many neurophysiological studies [7, 8]. A brain magnetic resonance imaging (MRI) study on patients with chronic TTH (≥ 15 headache days/month) demonstrated a gray matter (GM) decrease in the anterior cingulate, insula, orbitofrontal cortex, dorsal pons, and other structures of the pain processing network [9]. These structural changes have also been documented in patients with migraine [10–13]. TTH and migraine thus seem more inter-related than would be suggested by their diagnostic criteria.

However, the findings suggesting a common pathophysiology for TTH and migraine must be confirmed without interference from migraine comorbidity. Notably, a study reported normal interictal plasma levels of calcitonin gene-related peptide (CGRP) in patients with chronic TTH; however, in the patient subgroup with pulsating pain quality, the CGRP level was higher, as in the group of patients with interictal migraine [14]. These findings implied that the presence of even one migrainous feature in patients with TTH may eventually lead to an association with migraine in the pathophysiology. In addition, some studies on quantitative sensory testing [15], brainstem excitability [16], laser evoked potentials [17], and temporal discrimination thresholds [18] have congruently revealed different somatosensory information processing between TTH and migraine, although migraine comorbidity was not deliberately excluded in patients with TTH. The present study thus hypothesized that TTH and migraine are different in brain morphology, which may reflect their distinct symptomatology, sensory processing, and disease burden. To test this hypothesis, we used strict criteria to enroll patients with “pure” TTH instead of relatively loose criteria, as suggested by ICHD-III beta (see Methods for details). Brain morphology was analyzed using voxel-based morphometry (VBM), a technique that has been widely employed to evaluate brain morphological alternations in various chronic pain syndromes including migraine [19]. To our knowledge, two MRI studies [9, 20] have employed VBM to investigate the brain morphological change in TTH. A study on episodic TTH (<15 headache days/month) [20] did not reveal any GM change, whereas a study on chronic TTH, as aforementioned, suggested reduced GM in the pain processing network [9]. Few studies have compared the brain morphological differences between TTH and migraine. Clarifying these issues may facilitate the development of a TTH-specific therapy or brain signature.

## Methods

### Participants

Consecutive patients with episodic or chronic TTH were recruited from the Headache Clinic of Taipei Veterans General Hospital. For data comparison, this study also enrolled patients with episodic migraine (without aura) and chronic migraine, and healthy controls. The diagnosis of episodic migraine (*code 1.1*) and chronic migraine (*code 1.3*) was made according to the ICHD-III beta criteria [3]. Episodic and chronic TTH were also diagnosed based on the ICHD-III beta criteria, but a strict version was used instead—all patients were required to fulfill all (rather than ≥2) of the following four headache characteristics, which are defined as the core syndromes of TTH: bilateral, mild-to-moderate intensity, non-pulsating and not aggravated by routine physical activity. Moreover, patients were required to report no migrainous features (nausea, vomiting, photophobia, or phonophobia) associated with their headaches, although the original ICHD-III beta criteria allow for the presence of either photophobia or phonophobia. Patients with episodic TTH reported 1–14 headache days/month (i.e., frequent episodic TTH, code 2.2), whereas patients with chronic TTH (code 2.3) experienced ≥15 headache days/month for at least 3 months. Patients fulfilling the criteria of medication overuse headache (*code 8.2*) were excluded. Healthy controls did not have past or family histories of headache or any headache attacks in the previous year. All participants were right-handed, denied any history of systemic or neurologic disease, and presented with normal physical and neurological examinations. Participants who used any medications (including headache prophylactic drugs) or hormone therapy daily before participation were excluded. The hospital’s Institutional Review Board approved the study protocol and each participant provided written informed consent.

At the first visit, all patients completed a semi-structured questionnaire on their demographics and headache profiles. They also completed a headache diary for at least 3 months after recruitment. The Migraine Disability Assessment (MIDAS) questionnaire assessed headache-related disability [21]. Depression was evaluated using the Beck Depression Inventory (BDI) [22].

Each participant underwent a scheduled MRI examination during the interictal period, which was defined as the absence of any headache within the 2 days before (days −1 and −2) and after (days +1 and +2) the MRI examination (day 0). The MRI examination was re-scheduled when an acute headache attack occurred during this period, or when analgesics, triptans, or ergots were used for any reason within the 48 h preceding the examination. Notably, the presence of background or interval headache during the defined interictal period was permitted in patients with chronic migraine. For patients with chronic TTH, we did not synchronize all the MRI examinations in the interictal periods because some patients experienced almost daily or continuous headaches, and interictal imaging was thus impracticable. The temporal relationship between MRI examination and headache attacks was determined through the headache diary and follow-up phone calls.

### MRI protocol

All participants underwent a three-dimensional whole-brain T1-weighted anatomical scan on a 3.0 T whole-body MRI scanner (Siemens Magnetom Tim Trio, Erlangen, Germany) equipped with a 32-channel phase array head coil at National Yang-Ming University, Taipei, Taiwan. For the acquisition of T1-weighted anatomical images, a sagittal multi-planar rapid acquire gradient echo sequence was employed with the following scanning parameters: repetition time = 2530 ms, echo time = 3.0 ms, inversion time = 1100 ms, flip angle = 7°, 192 sagittal slices (without inter-slice gap and interpolation), number of excitations = 1, field-of-view = 224 × 256 mm^2^, matrix size = 224 × 256, and isotropic 1.0 mm^3^ resolution. All images were acquired parallel to the plane connecting the anterior and posterior commissure, and were visually assessed for image artifacts and significant motion problems.

### VBM analysis of brain GM

Voxel-wised GM volume estimates were calculated using the VBM analysis framework [23] with the VBM8 toolbox (version 445, http://dbm.neuro.uni-jena.de/vbm.html), Statistical Parametric Mapping software (SPM8 version 6313, Wellcome Institute of Neurology, University College London, UK, http://www.fil.ion.ucl.ac.uk/spm/) and Matlab R2010a (Mathworks, Natick, MA, USA). The image preprocessing pipeline has been detailed in other studies [24, 25]. Before tissue segmentation, the image origin was set automatically using the center of mass approach with the VBM8 toolbox. This step reduced between-subject variability that may have confounded the subsequent tissue segmentation and image registration. Then, the native-space T1 anatomical images were corrected for field inhomogeneity and further partitioned into GM, white matter and cerebrospinal fluid compartments. To account for differences in global brain volume across the study participants, individual native-space tissue segments were further affine-aligned into the Montreal Neurological Institute (MNI) space. For inter-subject registration, these affine-aligned GM and white matter images were warped to a study-specific tissue template constructed from all study participants using a diffeomorphic non-linear image registration algorithm [26]. Subsequently, the normalized GM tissue images were modulated by the Jacobian determinants of the deformation field to preserve the local tissue volume estimation during the non-linear image deformation process. These modulated GM tissue images were spatially smoothed using an isotropic Gaussian kernel with 8-mm full-width at half-maximum and served as the inputs for the voxel-wise statistical analyses. To create the explicit mask for the voxel-wise statistical analyses, unmodulated GM images in the MNI space were also obtained and averaged across all the study participants. In construction of the final consensus mask, the voxels with GM tissue probabilities lower than a threshold value of 0.2 were excluded to minimize potential edge effects between different tissue types.

### Statistical analysis of neuroimaging data

Voxel-wise statistical analyses and region of interest (ROI) analyses were conducted using the generalized linear model Flex toolbox (http://mrtools.mgh.harvard.edu/index.php?title=GLM_Flex) and SPSS software (version 17, SPSS, Chicago, IL, USA) respectively. For all voxel-wise statistical analyses, the results revealed significant effects at the cluster-level family-wise-error corrected *P* value of <0.05, with a cluster forming threshold of a voxel-level *P* value of <0.005, and 271 voxel extents. This statistical criterion was determined based on the empirical results of a Monte Carlo simulation using 3dClustSim (permutations = 10,000; with explicit GM mask; version AFNI_17.1.04). In addition to the thresholded results reported as the major findings in this manuscript, we have also uploaded all the un-thresholded statistical maps to the NeuroVault website, available through the following permanent link: https://neurovault.org/collections/3198/. In this study, preprocessed whole-brain GM tissue segments and the mean GM volumes of specific ROIs were used to address the following three research questions:

#### Question 1: Is the GM volume differed in patients with TTH and migraine (vs. controls) and linked to headache profiles?

To determine the GM volume difference between controls and patients with TTH, or migraine, a statistical design of voxel-wise single-factor three-level (TTH, migraine, and controls) analysis of covariance (ANCOVA) was employed, with age, sex, and BDI entered as nuisance variables. There were four contrasts for this statistical design: controls > TTH; controls < TTH; controls > migraine; controls < migraine. To investigate the clinical relevance, the GM volumes of the clusters with a significant between-group main effect (TTH vs. controls or migraine vs. controls) were further extracted, averaged, and correlated with headache profile (TTH or migraine, dependent on the contrast) using Spearman’s rank order correlation. Participants’ age, sex, and BDI were also included as confounding covariates in the correlation analyses.

#### Question 2: Is the GM volume different between TTH and migraine and modulated by headache frequency?

A voxel-wise 2 × 2 factorial design with the factors TYPE (TTH vs. migraine) and FREQUENCY (episodic vs. chronic headache) was used to examine the main effects of TYPE and FREQUENCY and their interaction. Age, sex and BDI were also entered as covariates of no interest. In the post-hoc analysis, the GM volume was compared between TTH and migraine in their episodic forms (episodic TTH vs. episodic migraine) and chronic forms (chronic TTH vs. chronic migraine), respectively.

#### Question 3: Is GM difference predictive of the headache type?

The GM volumes indicating group differences between headache diagnoses (TTH vs. migraine) were further analyzed using a logistic regression model which adjusted for age, sex, and BDI to confirm the significance of the headache type prediction (TTH vs. migraine). The predictive value of the regression model was further estimated using the area under the receiver operating characteristic (ROC) curve.

The threshold for statistical significance was a *P* value of <0.05 (two-tailed) throughout the study.

## Results

### Demographics and clinical profiles

A total of 156 individuals participated in this study: 43 controls, 56 patients with migraine (31 with episodic and 25 with chronic migraine), and 57 patients with TTH (30 with episodic and 27 with chronic TTH). Of the 57 patients with TTH, eight (five with episodic and three with chronic TTH) were excluded because of the presence of migrainous features according to their headache diary. The remaining 49 patients (25 with episodic and 24 with chronic TTH) became our final TTH group. The three groups did not differ in age and sex; however, BDI score was higher in the migraine (*P* < 0.001 vs. controls) and TTH (*P* = 0.020 vs. controls) groups (Table 1). Patients with migraine and TTH did not differ in average disease duration or headache frequency, but headache intensity, MIDAS score, and analgesics use frequency—as expected—were all higher in the migraine group than in the TTH group (all *P* < 0.05). The demographics and clinical parameters of the five participant groups (controls vs. episodic migraine vs. chronic migraine vs. episodic TTH vs. chronic TTH) are shown in Additional file 1: Table S1.

**Table 1:** Demographics and clinical profile of the three participant groups

	Group		
	Control (*n* = 43)	Migraine (*n* = 56)	TTH (*n* = 49)
Age	36.2 ± 7.7	37.5 ± 7.6	39.0 ± 12.0
Gender	28F/15M	37F/19M	26F/23M
Episodic/chronic	–	31/25	25/24
Headache frequency (d/mo)	–	13.8 ± 10.5	14.0 ± 10.6
Disease duration (mo)	–	194.6 ± 116.7	156.1 ± 144.5
Headache intensity (0–10) ^a^	–	5.9 ± 2.1	3.5 ± 1.3
MIDAS (0–270) ^a^	–	26.1 ± 35.8	8.9 ± 16.8
BDI (0–63) ^b,c^	4.2±4.8	8.7± 5.7	7.3 ± 5.0
Analgesics use profile			
Frequency (d/mo) ^a^	–	4.4 ± 2.5	1.6 ± 2.5
Types of analgesics (% of patients)			
Simple analgesics		16.1%	12.2%
Compound analgesics		5.4%	4.0%
NSAIDs		8.9%	6.1%
Ergots		5.4%	0%
Triptans		5.4%	0%

*BDI* Beck Depression Inventory, *d* days, *MIDAS* migraine disability assessment, *mo* month, *NSAIDs* Nonsteroidal anti-inflammatory drugs, *TTH* tension-type headache

^a^*p* < 0.05 for migraine vs. TTH

^b^*p* < 0.05 for migraine vs. control

^c^*p* < 0.05 for TTH vs. control

### Altered GM volume in patients with TTH and migraine (vs. controls)

**Fig. 1:**
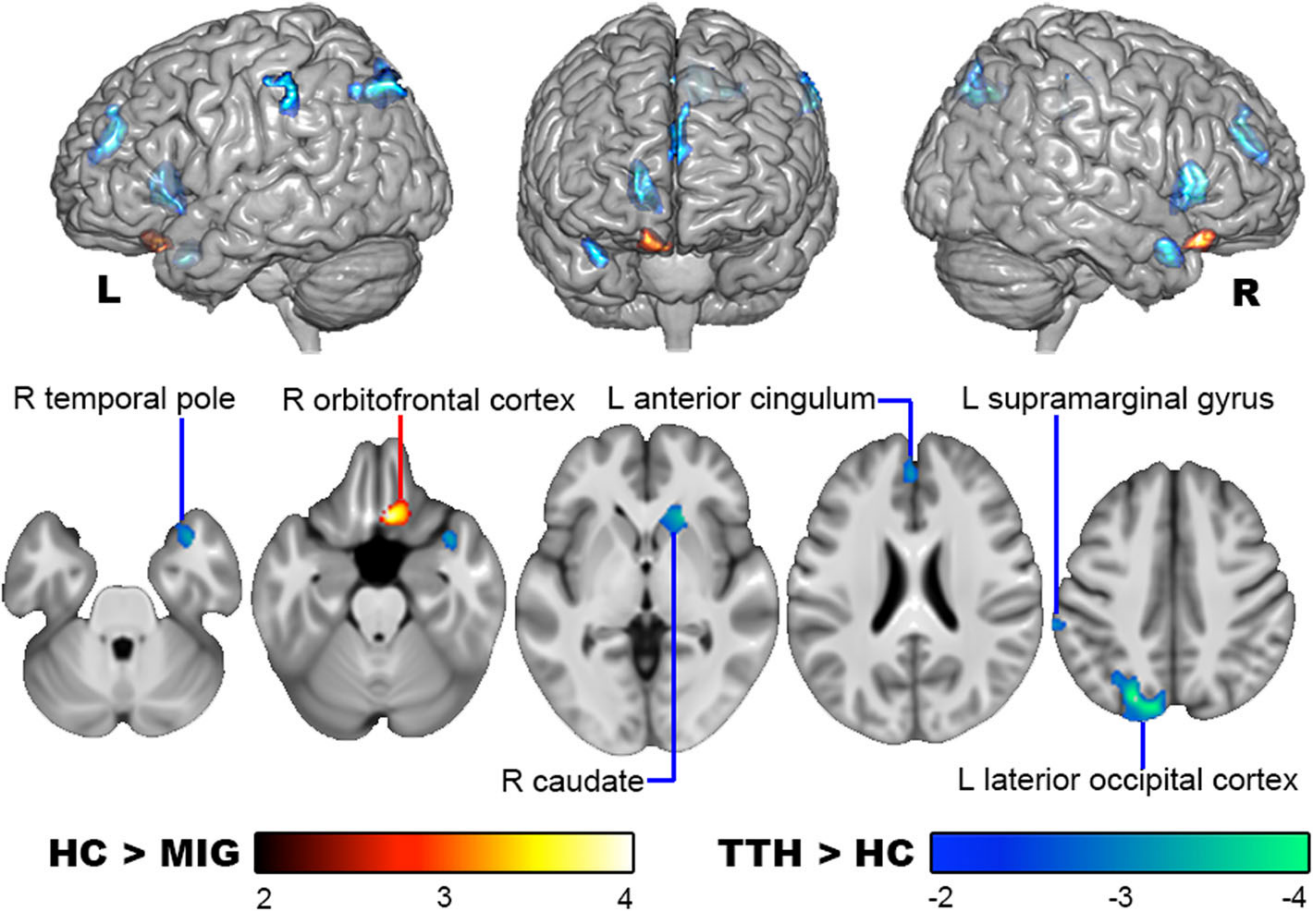
Altered gray matter volume in TTH and migraine. In comparison with controls, the patients with TTH exhibited a gray matter volume increase in the anterior cingulate cortex, supramarginal gyrus, temporal pole, lateral occipital cortex, and caudate. The patients with migraine conversely exhibited a gray matter volume decrease in the orbitofrontal cortex. HC: healthy controls; L: left; MIG: migraine; R: right; TTH: tension-type headache

GM volume was increased in patients with TTH whereas it was decreased in patients with migraine compared with the controls (Table 2 and Fig. 1). In patients with TTH, GM volume was increased in the right caudate, temporal pole, left anterior cingulate cortex, supramarginal gyrus, and lateral occipital cortex. In the migraine group, GM volume was decreased only in the right orbitofrontal cortex. In the subgroup analysis, GM volume (vs. controls) was unaltered in the episodic and chronic migraine groups, but was increased in specific brain regions in patients with episodic TTH (the right caudate–putamen, temporal pole, cerebellum, left anterior cingulate cortex, superior and middle frontal gyrus, and lateral occipital cortex) and chronic TTH (the right caudate, left supramarginal gyrus, and lateral occipital cortex) (Additional file 2: Figure S1). None of the aforementioned GM changes were correlated with headache profile.

**Table 2:** Altered gray matter volume in patients with migraine and TTH

MNI coordinates			Cluster size	Anatomical region	Local peak *T*-value
x	y	z			
Migraine < controls					
6	23	−24	287	R orbitofrontal cortex	4.05
TTH > controls					
-11	−80	51	1316	L lateral occipital cortex	−5.27
20	24	3	628	R caudate	−3.70
36	12	−27	278	R temporal pole	−3.51
-60	−33	41	295	L supramarginal gyrus	−3.37
-2	42	29	426	L anterior cingulate cortex	−3.28

*Abbreviations*: *L* left, *MNI* the Montreal Neurological Institute, *R* right, *TTH* tension-type headache

### Altered GM volume in different headache types (TTH vs. migraine) and frequencies (episodic vs. chronic)

**Fig. 2:**
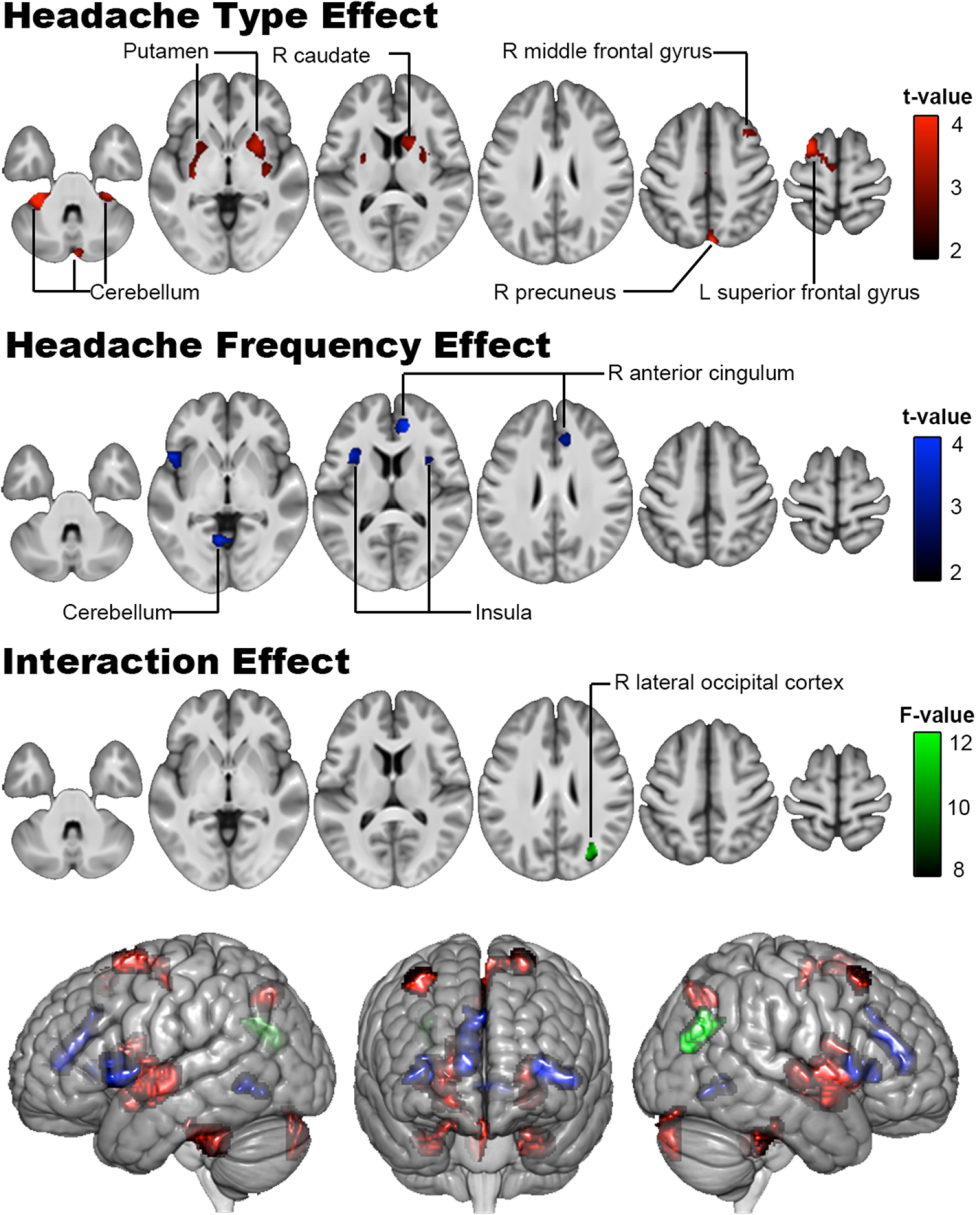
Difference of gray matter volume between TTH and migraine. A 2 × 2 ANCOVA analysis was used to investigate the effects of headache types (TTH vs. migraine), headache frequency (episodic vs. chronic) and their interaction upon the gray matter volume difference between TTH and migraine. The brain regions with gray matter differences are color-coded in red (TTH > migraine), blue (episodic > chronic) and green (type × frequency interaction). L: left; MIG: migraine; R: right; TTH: tension-type headache

A voxel-wise 2 × 2 factorial analysis revealed the significant effects of headache type, frequency, and their interaction in the comparison of GM volume between patients with TTH and migraine (Table 3 and Fig. 2). In the effect of headache type (TTH vs. migraine), GM volume was lower for the migraine group in the bilateral putamen, cerebellum, right caudate, putamen, precuneus, middle frontal gyrus, and left superior frontal gyrus. Regarding the effect of headache frequency (episodic vs. chronic), GM volume was lower for the chronic group (TTH and migraine combined) in the bilateral insula, right anterior cingulate cortex, and cerebellum. A significant headache type × frequency interaction was discovered in the right lateral occipital cortex.

**Table 3:** A 2 × 2 ANCOVA analysis for the gray matter volume difference between TTH and migraine

MNI coordinates			Cluster size	Anatomical region	Local peak T/F-value
x	y	z			
***Headache type effect (TTH > migraine)***					
-27	−38	−39	705	L cerebellum VI	4.57
5	−74	50	380	R precuneus cortex	4.38
-24	8	65	611	L superior frontal gyrus	4.23
3	−87	−26	590	R cerebellum crus II	4.03
32	−17	6	1839	R putamen	3.81
32	−33	−33	340	R cerebellum V	3.75
34	18	59	324	R middle frontal gyrus	3.55
-29	−20	3	1128	L putamen	3.55
17	13	8	638	R caudate	3.45
***Headache frequency effect (Episodic > Chronic)***					
14	45	15	933	R anterior cingulate cortex	3.96
-47	17	0	1070	L insula	3.87
-6	−57	−2	366	L cerebellum V	3.78
36	29	7	364	R insula	3.54
***Type × frequency interaction effect***					
29	−72	24	530	R lateral occipital cortex	14.04
***Post-hoc comparisons***					
*Episodic TTH > Episodic migraine*					
26	14	1	1850	R putamen	4.21
3	−86	−21	542	R cerebellum crus I	3.91
41	26	51	573	R middle frontal Gyrus	3.91
-29	−18	0	1131	L putamen	3.87
9	12	14	364	R caudate	3.30
*Episodic TTH < episodic migraine*					
42	−78	−12	535	R lateral occipital cortex	−3.55
*Chronic TTH > Chronic migraine*					
-27	−41	−38	640	L cerebellum VI	3.67
*Chronic TTH < Chronic migraine:* non-significant					

*Abbreviations*: *L* left, *MNI* Montreal Neurological Institute, *N.S.* non-significant, *R* right, *TTH* tension-type headache

**Fig. 3:**
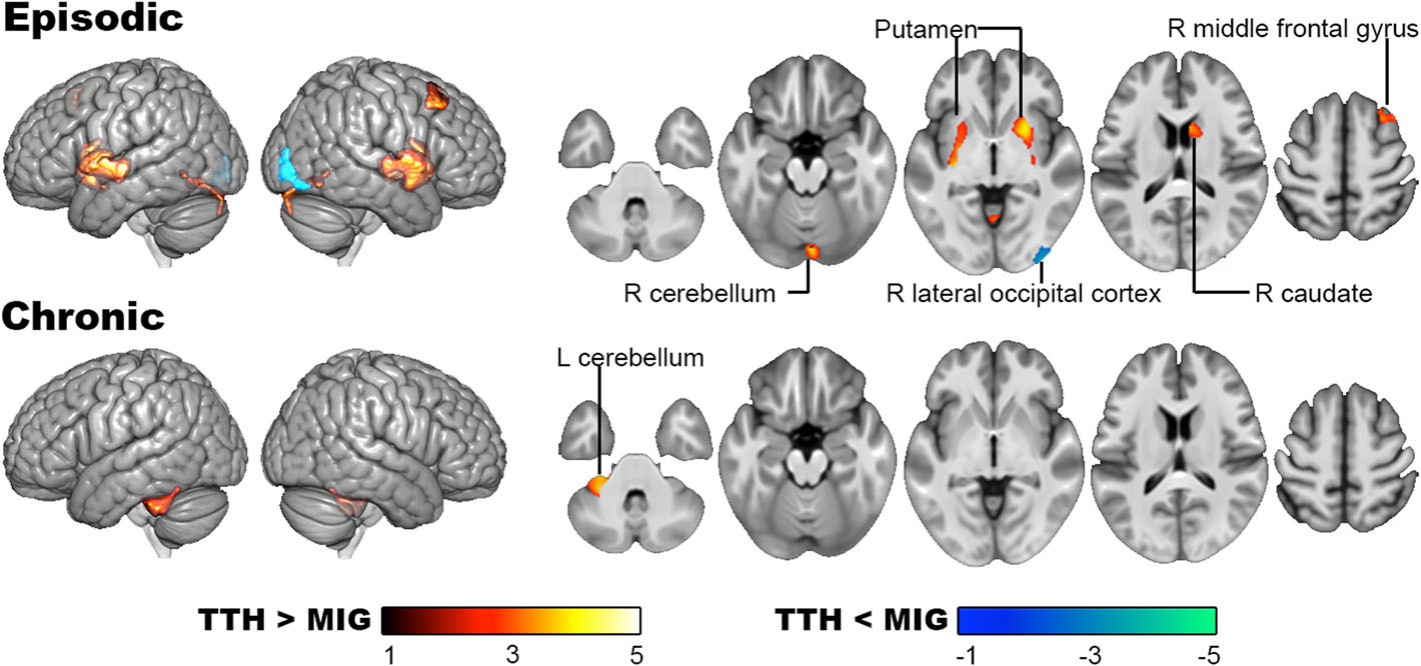
Post-hoc analysis for the gray matter volume difference between TTH and migraine (2 × 2 ANCOVA). The episodic TTH vs. episodic migraine groups demonstrated a higher gray matter volume in the bilateral putamen, right caudate, middle frontal gyrus, and cerebellum. Conversely, gray matter volume of the right lateral occipital cortex was lower in episodic TTH compared with episodic migraine. A comparison of gray matter volume between chronic TTH and chronic migraine only revealed a higher gray matter volume of the left cerebellum in chronic TTH. L: left; MIG: migraine; R: right; TTH: tension-type headache

In the post-hoc analysis (Table 3 and Fig. 3), the episodic TTH vs. episodic migraine groups demonstrated a higher GM volume in the bilateral putamen, right caudate, middle frontal gyrus, and cerebellum. Conversely, GM volume of the right lateral occipital cortex was lower in episodic TTH compared with episodic migraine. A comparison of the GM volume between chronic TTH and chronic migraine only revealed a higher GM volume of the left cerebellum in chronic TTH.

### Predictive value of GM volume difference for headache types

**Fig. 4:**
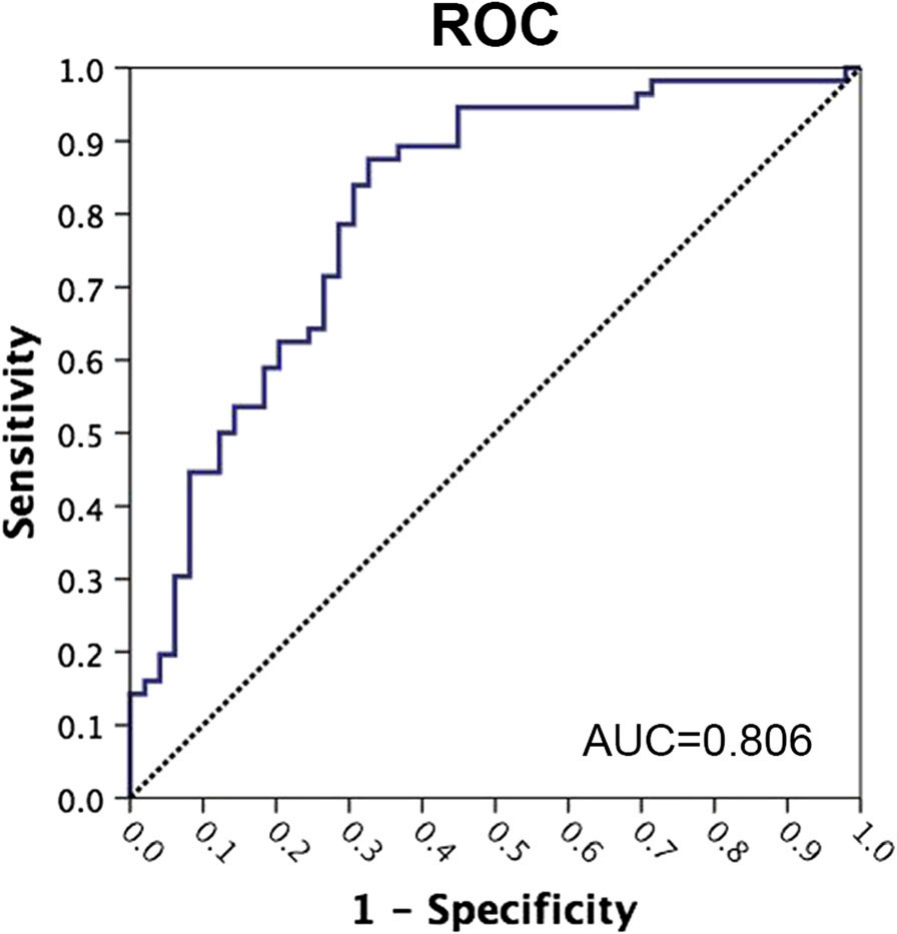
Receiver operating characteristic (ROC) analysis to distinguish TTH from migraine. In ROC analysis, the gray matter volumes of the left superior frontal gyrus and right cerebellum V combined had excellent discriminative ability for distinguishing TTH and migraine (area under the curve [AUC] = 0.806)

A logistic regression model was employed to assess whether the GM volume differences between TTH and migraine could predict headache types (TTH vs. migraine). After adjustment for age, sex, and depression, two of the brain regions indicating a headache-type effect (TTH > migraine; Table 3 and Fig. 2) were predictive of TTH diagnosis: the left superior frontal gyrus (beta = 15.92, *P* = 0.001) and right cerebellum V (beta = 10.33, *P* = 0.006). In the ROC analysis, to distinguish TTH from migraine, the area under the curve of the regression model was 0.806, indicating good discrimination (Fig. 4).

## Discussion

Structural MRI and VBM were used to compare GM volume between migraine and “strict-criteria” TTH. We determined that TTH and migraine differed in brain morphology because (1) the GM volume of specific brain regions were increased (anterior cingulate cortex, supramarginal gyrus, temporal pole, lateral occipital cortex, and caudate) in patients with TTH whereas decreased (orbitofrontal cortex) in patients with migraine compared with healthy controls; (2) a direct comparison of GM volume between both headache disorders revealed a lower GM volume in the superior and middle frontal gyri, cerebellum, dorsal striatum (putamen and caudate), and precuneus in patients with migraine; (3) the GM of the left superior frontal gyrus and right cerebellum V together demonstrated good discriminative ability for TTH and migraine in the ROC analysis. In addition, headache chronification in patients with TTH and migraine was associated with a GM decrease in the bilateral insula and anterior cingulate cortex. Although the neurobiological basis for these GM plastic changes is not sufficiently understood, we discuss herein their potential relevance for TTH and migraine pathophysiology.

### GM increase in TTH and decrease in migraine (vs. controls)

Earlier MRI studies on TTH have revealed no volumetric GM change in episodic TTH [20] and reduced GM in chronic TTH in the anterior cingulate, insula, orbitofrontal cortex, dorsal pons, and other brain structures involved in pain processing [9]. Our findings of increased GM volume in TTH (also in episodic and chronic subgroups) appear to conflict with earlier studies, although the brain regions that exhibited a GM increase are also part of the pain processing network [27]. In addition to the adjustment for the depression effect and different *P* value thresholds, the strict TTH criteria employed in this study may be the main reason for this discrepancy. For patients with migraine, this study was in line with the literature [10, 12] indicating reduced GM in the orbitofrontal cortex, a region associated with reward, adaptive behavior, and pain processing [28]. Notably, most migraine studies on GM change, despite heterogeneous findings in localization, have congruently demonstrated reduced GM in the pain processing network [10, 11, 13, 29–31].

### GM difference between TTH and migraine

One notable finding of this study was that TTH and migraine differed in the GM volumes of several prefrontal and cerebellar regions (Fig. 2), and both types of headaches could be distinguished by the volumes of the left superior frontal gyrus and right cerebellum V in ROC analysis. In meta-analyses, the prefrontal cortex was identified as being the most crucial brain area associated with structural change in migraine [32, 33]. A discriminative analysis of migraine without aura (vs. controls) also identified, using a machine learning classifier, the superior frontal gyrus as one of the most discriminative GM features [34]. The present finding of a lower prefrontal GM volume in patients with migraine has three clinical implications. First, the prefrontal cortex is associated with the descending inhibitory mechanism of pain modulation [35]. A GM decrease in this region may be linked to inhibitory dysfunction and hence a heightened severity of migraine versus TTH. Second, the superior and middle frontal gyri are involved in task monitoring and temporal organization, two crucial aspects of executive function [36]. GM decrease in these prefrontal regions was linked to executive dysfunction (i.e., delayed response time to task set-shifting) in patients with migraine [37]. Thus, lower prefrontal GM in patients with migraine may reflect a greater extent of cognitive dysfunction in migraine versus TTH [38]. Third, a higher prevalence of psychiatric comorbidities in patients with migraine versus those with TTH [39] may also partly be explained by the lower prefrontal GM in patients with migraine. The discriminative ability of cerebellum for TTH and migraine appears to correspond with most studies that indicated a GM decrease [31, 33, 40] and subclinical dysfunction [41–44] of the cerebellum in patients with migraine. The cerebellum also plays an inhibitory role in nociception, given its extensive connection with the prefrontal cortex [45]. Thus, the relative GM decrease in the cerebellum may be partly related to heighted pain severity in migraine.

The dorsal striatum and precuneus also differed between patients with TTH and migraine in GM volume. The dorsal striatum is part of the pain processing network, and its activation may encode pain intensity [46]. The difference in striatal volume between TTH and migraine may be explained by a GM increase in patients with TTH (vs. controls, Fig. 1) and a tendency for migraine to reduce GM in this region [29, 47]. The precuneus is a pivotal region of the default mode network, which was particularly sensitive to the cognitive states in self-referential tasks [48]. The functional connectivity of the default mode network was changed in various pain conditions [49], and the connectivity of the network with insula has been reported to encode pain intensity [50, 51]. The relative GM decrease in migraine (vs. TTH) is thus explicable by the extent to which migraine interferes with information processing and intrinsic variation in brain activity. We are not sure why the lateral occipital cortex is increased in GM in episodic migraine (vs. episodic TTH) and in TTH (vs. controls). However, this brain region, in addition to its well-known involvement in visual processing, is associated with cognitive evaluation of pain [52], and has been linked to abnormal emotional processing and self-focused attention [53]. An earlier MRI study on episodic migraine also showed increased cortical thickness and gyrification index in this region [54].

### GM change associated with headache frequency

That the GM of the anterior cingulate cortex and insula decreases as a headache evolves from the episodic to chronic form is not unexpected (headache frequency effect). The anterior cingulate cortex and insula are the pivotal relay regions of the pain network and involved in the affective and cognitive processing of subjective pain experience [27, 46]. Notably, their GM decreases are not specific to chronic headaches but have also been reported in various types of chronic pain including fibromyalgia [55], classical trigeminal neuralgia [56], and phantom limb pain [57]. These plastic changes may underpin the common emotional distress and cognitive dysfunction that cause disability in patients with chronic pain [58]. Moreover, the findings of this study suggest that the structural difference between TTH and migraine, as judged by the number of brain regions (Fig. 3), is more prominent in their episodic forms than chronic forms. The trend toward a structural similarity in the chronification of TTH and migraine may resemble the clinical scenario, in which chronic migraine often loses its vegetative characteristics (accompanying photophobia, phonophobia, nausea, vomiting, and headache exacerbation with physical exercise) and thus resembles TTH [59].

### GM change and allostatic load

The mechanism of GM volume change is beyond the scope of this study. However, the present findings of GM change in TTH and migraine (vs. controls) and the GM difference between both types of headaches mostly involved brain regions of the pain processing network, which suggested these plastic changes may reflect the allostatic load in response to headache pain [60]. It is posited that the pain processing network may present an adaptive volume increase to mild-to-moderate TTH pain, whereas a maladaptive volume decrease to the moderate-to-severe migraine pain. Notably, a pain severity-dependent plastic change in GM volume has been reported in chronic migraine and phantom limb pain [61, 62]. Earlier evoked potential studies that showed a deficient habituation in migraine in contrast with a relatively intact habituation in TTH also suggest a greater allostatic load in migraine (vs. TTH), hence a maladaptive brain response [63].

### Study limitations

This study was limited in terms of the generalizability of its findings to patients with migraine with aura or medication overuse and patients with TTH diagnosed according to the standard ICHD-III beta criteria. Moreover, our findings cannot be generalized to the ictal imaging data because dynamic GM change across the ictal–interictal cycle has been reported in TTH [20] and migraine [64]. The technique of VBM has inherent limitations [65]. Histological measures such as neuronal density do not correlate with VBM GM probability maps [66]. Changes in cerebral blood flow may apparently change GM volume in VBM analyses [67]. Our findings did not include areas that were specifically activated in migraine (i.e., the brainstem and hypothalamus [68]). However, the negative finding does not imply that the brainstem and hypothalamus have no role in differentiating between TTH and migraine in brain morphology, because these brain regions are relatively small for a whole-brain analysis. The complex tissue pattern in these deep structures may also hamper precision of the VBM-based tissue segmentation. Further 3 T MRI studies using methods refined for analyzing these specific structures are warranted to draw conclusions. Limited by its design, the present study could not elucidate the causal relationship between brain morphological change and headache phenotypes. A longitudinal study in the same patient group would be particularly valuable to examine whether the GM difference between TTH and migraine is a steady phenotypic marker independent of disease duration. Finally, the diagnostic ability of the GM volume to differentiate between TTH and migraine must be confirmed in a new patient population.

## Conclusions

TTH and migraine are separate headache disorders with different characteristics of GM change. The major morphological difference between the two types of headaches is a relative GM decrease in the prefrontal and cerebellar regions in migraine, which may reflect a higher allostatic load associated with this disabling headache. Our findings may facilitate the development of a TTH-specific treatment and phenotypic marker. However, these GM changes remain undetermined in the neurobiological mechanism, temporal stability, and causal relationship with headache phenotypes.

## Additional files

Additional file 1: Table S1. Demographics and clinical profile of the five participant groups (DOCX 17 kb)

Additional file 2: Figure S1. Altered gray matter volume in episodic and chronic TTH. In the subgroup analysis, gray matter volume (vs. controls) was unaltered in the episodic and chronic migraine groups, but was increased in specific brain regions in patients with episodic and chronic TTH. CTTH: chronic tension-type headache; ETTH: episodic tension-type headache; HC: healthy controls; L: left; R: right. (TIFF 2705 kb)

## Abbreviations

ANCOVA: Analysis of covariance
BDI: The Beck Depression Inventory
CGRP: Calcitonin gene-related peptide
GM: Gray matter
ICHD: International Classification of Headache Disorders
MIDAS: Migraine Disability Assessment
MNI: The Montreal Neurological Institute
MRI: Magnetic resonance imaging
ROC: Receiver operating characteristic
ROI: Region of interest
TTH: Tension-type headache
VBM: Voxel-based morphometry
