# Supplementary material for: Comparison of gray matter volume between migraine and “strict-criteria” tension-type headache
Source: J Headache Pain. 2018 Jan 15;19(1):4. doi: 10.1186/s10194-018-0834-6 (PMC5768588; doi:10.1186/s10194-018-0834-6)
Supplement: Supplementary file 1 — Demographics and clinical profile of the five participant groups (DOCX 17 kb) [file 10194_2018_834_MOESM1_ESM.docx]

**Additional file 1: Table S1.** **Demographics and clinical profile of the five participant groups**

|  | Group | | | | |
| --- | --- | --- | --- | --- | --- |
|  | Control  (n=43) | Episodic migraine  (n=31) | Chronic  migraine  (n=25) | Episodic  TTH  (n=25) | Chronic TTH  (n=24) |
| Age | 36.2±7.7 | 37.7±7.2 | 37.2±8.1 | 39.1±12.3 | 38.8±11.9 |
| Gender | 28F/15M | 19F/12M | 18F/7M | 13F/12M | 13F/11M |
| Disease duration (mo) | - | 200.1±124.7 | 187.7±108.1 | 174.2±144.6 | 137.2±145.0 |
| Headache frequency (d/mo) | - | 5.2±3.4 | 24.6±4.2 | 5.1±3.8 | 23.3±6.6 |
| Headache intensity (0-10) | - | 6.0±2.1 | 5.8±2.0 | 3.3±1.4 | 3.7±1.1 |
| BDI (0-63) | 4.2±4.8 | 7.4±5.9 | 10.5±5.2 | 6.0±4.8 | 8.7±4.9 |
| MIDAS (0-270) | - | 14.6±17.5 | 67.5±95.2 | 5.1±13.8 | 21.9±37.3 |
| Analgesics use frequency (d/mo) | - | 3.1±2.2 | 6.0±1.9 | 1.6±2.3 | 1.7±2.7 |

BDI: Beck Depression Inventory; d: days; MIDAS: migraine disability assessment; mo: months; TTH: tension-type headache.
