# Supplementary figures and images for: Comparison of gray matter volume between migraine and “strict-criteria” tension-type headache
Source: J Headache Pain. 2018 Jan 15;19(1):4. doi: 10.1186/s10194-018-0834-6 (PMC5768588; doi:10.1186/s10194-018-0834-6)

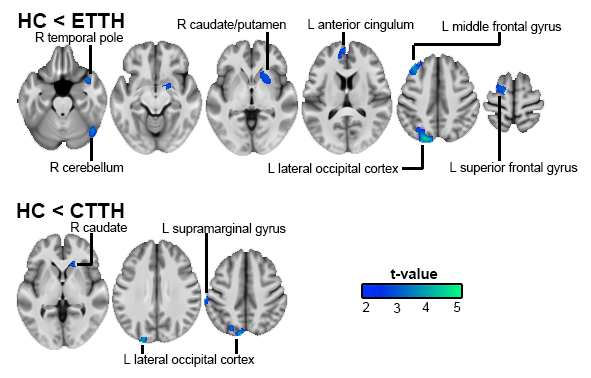

Supplement: Supplementary file 2 — Altered gray matter volume in episodic and chronic TTH. In the subgroup analysis, gray matter volume (vs. controls) was unaltered in the episodic and chronic migraine groups, but was increased in specific brain regions in patients with episodic and chronic TTH. CTTH: chronic tension-type headache; ETTH: episodic tension-type headache; HC: healthy controls; L: left; R: right. (TIFF 2705 kb) [file 10194_2018_834_MOESM2_ESM.tif]
